# Sequence analysis of cultivated strawberry (*Fragaria* × *ananassa* Duch.) using microdissected single somatic chromosomes

**DOI:** 10.1186/s13007-017-0237-8

**Published:** 2017-10-30

**Authors:** Tomohiro Yanagi, Kenta Shirasawa, Mayuko Terachi, Sachiko Isobe

**Affiliations:** 1 Faculty of Agriculture, Kagawa University, Miki-cho, Kita-gun, Kagawa 761-0795 Japan; 2 Kazusa DNA Research Institute, Kazusa-Kamatari, Kisarazu, Chiba Japan

**Keywords:** Strawberry (*Fragaria* × *ananassa* Duch.), Allopolyploid, Chromosome microdissection, Sequence analysis, Mapping

## Abstract

**Background:**

Cultivated strawberry (*Fragaria* × *ananassa* Duch.) has homoeologous chromosomes because of allo-octoploidy. For example, two homoeologous chromosomes that belong to different sub-genome of allopolyploids have similar base sequences. Thus, when conducting de novo assembly of DNA sequences, it is difficult to determine whether these sequences are derived from the same chromosome. To avoid the difficulties associated with homoeologous chromosomes and demonstrate the possibility of sequencing allopolyploids using single chromosomes, we conducted sequence analysis using microdissected single somatic chromosomes of cultivated strawberry.

**Results:**

Three hundred and ten somatic chromosomes of the Japanese octoploid strawberry ‘Reiko’ were individually selected under a light microscope using a microdissection system. DNA from 288 of the dissected chromosomes was successfully amplified using a DNA amplification kit. Using next-generation sequencing, we decoded the base sequences of the amplified DNA segments, and on the basis of mapping, we identified DNA sequences from 144 samples that were best matched to the reference genomes of the octoploid strawberry, *F.* × *ananassa*, and the diploid strawberry, *F. vesca*. The 144 samples were classified into seven pseudo-molecules of *F. vesca*. The coverage rates of the DNA sequences from the single chromosome onto all pseudo-molecular sequences varied from 3 to 29.9%.

**Conclusion:**

We demonstrated an efficient method for sequence analysis of allopolyploid plants using microdissected single chromosomes. On the basis of our results, we believe that whole-genome analysis of allopolyploid plants can be enhanced using methodology that employs microdissected single chromosomes.

**Electronic supplementary material:**

The online version of this article (doi:10.1186/s13007-017-0237-8) contains supplementary material, which is available to authorized users.

## Background

Cultivated strawberry (*Fragaria* × *ananassa* Duch.) is one of the most popular fruit crops worldwide and is grown across a wide range of regions from subarctic to tropical [1]. Cytogenetic studies have determined that the chromosome number of somatic cells of cultivated strawberry is 56 [2–5]. In addition, cultivated strawberry is an allo-octoploid, having three complex genome compositions: AABBBBCC [6], AAA′A′BBBB [7], or AAA′A′BBB′B′ [8]. In addition, Tennessen et al. [9] and Sargent et al. [10] have recently proposed updated models—AvAvB1B1B2B2BiBi and AA, bb, X–X, X–X, respectively. The amount of DNA within a haploid nucleus of cultivated strawberry has been estimated at 708–720 Mb [11]. On the basis of this data, the average DNA size of a single chromosome can be calculated as approximately 25–27.8 Mb. In addition, the mean chromosome length of some wild octoploid strawberries has been determined to be approximately 1 µm [12]. The size of a single chromosome in cultivated strawberry appears to be very small as likely as that in rice and *Arabidopsis thaliana*.

Allopolyploidy is a problem when conducting genetic analyses, because the presence of similar sub-genomes has led to multiple alleles and complex segregation ratios [13]. Thus, theoretical genetic analysis and breeding in cultivated strawberry are extremely difficult based Mendel’s law of inheritance. To resolve this problem, the determination of accurate base sequences covering the entire genome of cultivated strawberry is needed to construct a high-density linkage map. If this can be achieved, many DNA markers that follow Mendel’s law of inheritance could be discovered, and theoretical breeding using genomic selection could be performed with high reliability. Hirakawa et al. [14] reported the draft genome sequences of a Japanese cultivated strawberry using next-generation sequencing (NGS). The base sequences of entire DNA segments from the strawberry ‘Reiko’ were determined, and approximately 70% of these sequences were assembled into larger DNA scaffolds. However, to date, the sequences have not been assigned to chromosomes. Moreover, 30% of the genome remains unsequenced, because of the existence of sub-genomes that have homoeologous chromosomes. For example, two homoeologous chromosomes that belong to different sub-genomes may have similar but slightly different base sequences. Thus, when performing standard genome assembly, it is difficult to accurately assign such sequences to an appropriate pseudo-molecular chromosome. To overcome this problem, it is necessary to develop an alternative method of sequence analysis for allopolyploids.

In the present study, in order to avoid difficulties arising from the occurrence of homoeologous chromosomes, we attempted to conduct sequence analysis using microdissected single somatic chromosomes. Although a technique for chromosome microdissection was developed in the 1980s [15], it has been unpopular for elucidating the base sequences of whole genomes. Scalenghe et al. [16] initially microdissected a small segment of the chromosome in *Drosophila melanogaster*, and demonstrated the possibility of directly generating DNA segments. As discussed by Zhou and Hu [17], many studies have been conducted using chromosome microdissection for human and animal cells, but a smaller number have been performed in plants because chromosome sample preparation is more difficult in plants. In higher plants, Sandery et al. [18] first reported microdissection and DNA generation of B-chromosomes in rye. Subsequently, chromosome microdissection has been used in several facets of genomic research, including (1) genetic linkage map and physical map construction, (2) generation of probes for chromosome painting, and (3) generation of chromosome-specific expressed sequence tag libraries [19]. However, to date, few studies have used chromosome microdissection to determine base sequences of whole genomes in plants.

The purpose of the present study was to determine the effectiveness of sequence analysis using single chromosomes for a typical allopolyploid cultivated strawberry plant. Furthermore, we also examined the possibility of amplifying DNA from a very small single chromosome using a DNA amplification kit.

## Results and discussion

### Efficiency of the microdissection system

**Fig. 1:**
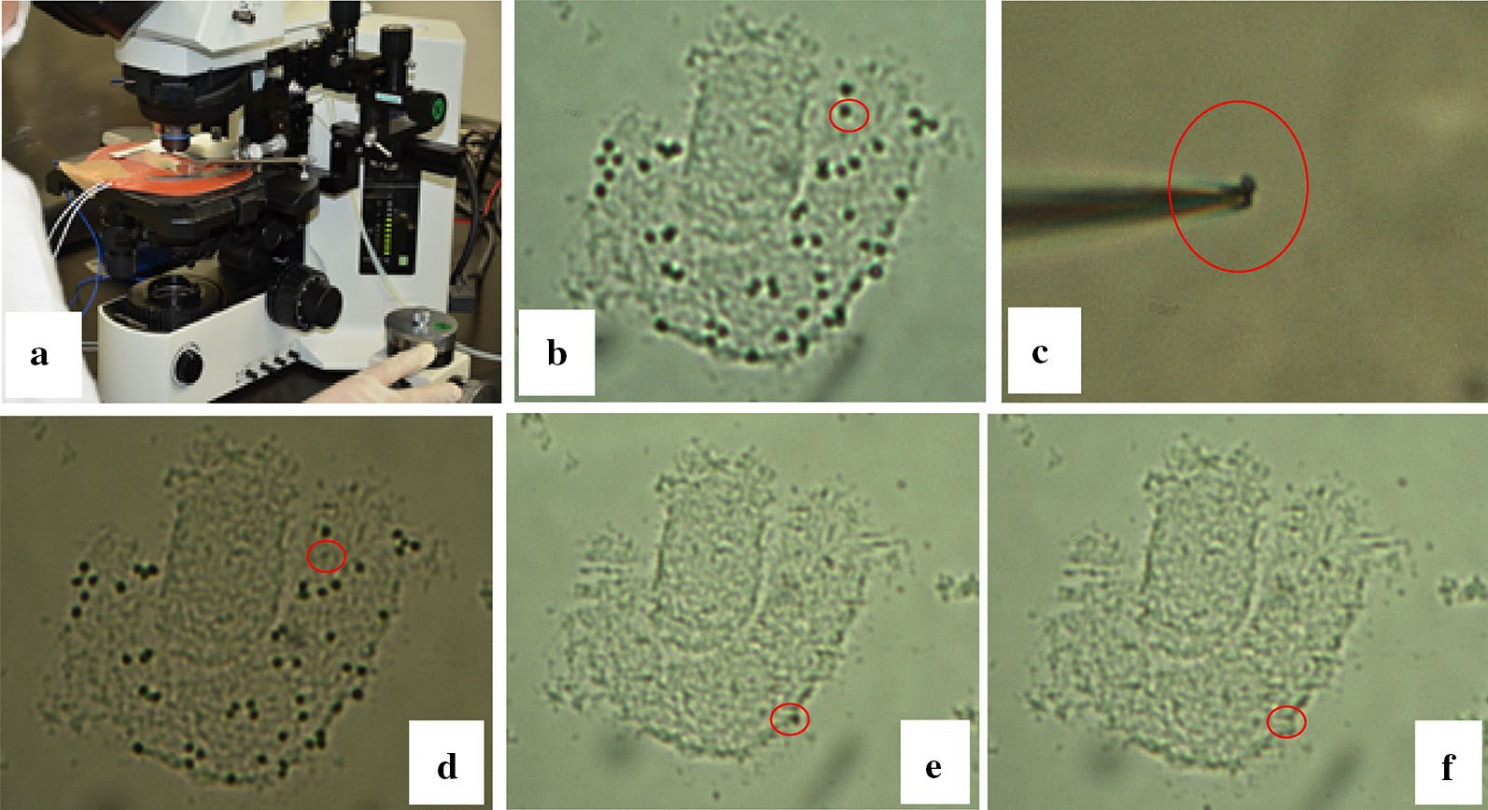
The microdissection equipment used in the present experiment and some pictures of chromosome and somatic cells of the cultivated strawberry. **a** Microdissection equipment which was used in the present experiment, **b** chromosome image of the cultivated strawberry; the red circle shows the chromosome which will be selected, **c** a single dissected chromosome on the glass needle, **d** the chromosome in **b** is disappeared, **e** the red circle shows the final chromosome of the somatic cell, and **f** all chromosomes were selected in the somatic cell

By way of illustrating chromosome microdissection in the cultivated strawberry, the equipment used and pictures of the chromosome and somatic cells are shown in Fig. 1. Fifty-four chromosomes were observed in somatic cell (Fig. 1b) under a light microscope, and used for microdissection. The remaining two chromosomes were missing. In Fig. 1c, a single microdissected chromosome can be seen at the terminal portion of a glass needle. The single chromosome surrounded by a red circle (Fig. 1b) subsequently disappeared (Fig. 1d), and the remaining 53 were individually selected (Fig. 1e). All chromosomes were eventually selected and removed from the somatic cell (Fig. 1f). Although the time from installing a new glass needle to placing a single chromosome in a PCR tube was not measured, on the basis of the total time of the microdissection and the number of chromosomes microdissected, we estimate that it probably took approximately 2 min to dissect a single chromosome. In general, chromosome microdissection can be performed using (1) a light microscope with a micromanipulator system and glass needle, and (2) a light microscope with a laser capture microdissection system. In the present study, a light microscope was used in conjunction with a micromanipulator system and glass needle. Using this system, because chromosome could be moved when it was pushed carefully by the apex of the glass needle, partially overlapped chromosomes could easily be separated and individually selected, which would not have been possible using a laser capture microdissection system. Further, a flow-cytometry and sorting devices can correct and take some same-sized chromosomes. However, using such a device might result in contamination from similar-sized homoeologous chromosomes because of its limited sensitivity, as indicated by Zhou and Hu [17]. Recently, Capal et al. [20] reported that it was possible to take one 3B chromosome of wheat by the flow cytometry and sorting devices. However, the paper did not show the method to confirm whether one chromosome was precisely selected in one tube or not. In addition, we thought that the chromosomes of the cultivated strawberry were very small and similar size unlike the chromosomes of wheat. For this reason, the flow-cytometry and sorting devices were not used in the present research. Chromosome microdissection with the micromanipulator system used in the present study generally requires an experienced operator; however, under the guidance of a person skilled in chromosome microdissection, a beginner can select a single chromosome after practicing just two or three times. Furthermore, since the size of cultivated strawberry chromosomes (approx. full length = 1 µm) appears to be at the lower limit for microdissection, chromosomes larger than those of cultivated strawberry can be selected using the same method.

### Confirmation of DNA amplification from a single chromosome of cultivated strawberry

**Fig. 2:**
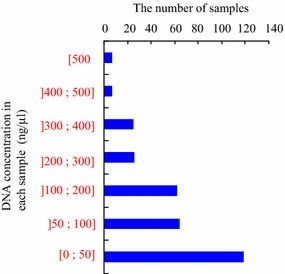
Frequency distribution of the amplified DNA concentration by the Illustra Single Cell GenomiPhi DNA Amplification kit. The 310 single chromosome were used for the DNA amplification

In the present study, 310 chromosomes were removed from 10 metaphase somatic cells of cultivated strawberry. To confirm the efficiency of the DNA amplification kit for amplifying single chromosome DNA of cultivated strawberry, the DNA concentration of each amplified sample was measured. The DNA concentration of the samples differed from almost zero to more than 500 ng/ µL (Fig. 2). Approximately 60% of the samples were amplified to more than 50 ng/µL.

**Fig. 3:**
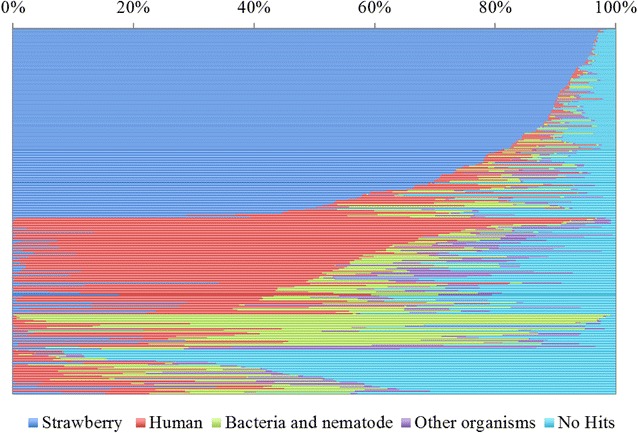
The results of mapping onto the reference genome of strawberry (FAN_r1.1 + *F. vesca*), human, bacteria, nematode and other organisms. The 1000 reads were extracted from the sequence data and mapped on the reference genome data. The 288 mapping data were summarized in this figure. Each thin bar exhibited the data of each sample

To clarify the components of each amplified DNA segment, the base sequences of the 288 samples with higher concentrations of DNA were decoded by NGS. For each sample, 1000 reads were extracted from the sequence data and mapped onto strawberry (FAN_r1.1 + *F*. *vesca*), human, bacterial, and nematode reference genomes, among others. The mapping results for the 288 samples are shown in Fig. 3. Of these, the amplified DNA segments from 144 samples had more than 50% of reads that matched the reference genome of strawberry. The maximum matched value of 983 reads was recorded for sample FaMD-4-A10. These results clearly demonstrated that the Illustra Single Cell GenomiPhi DNA Amplification kit could amplify the DNA sequence of the strawberry chromosome by following the manufacturer’s protocol, although DNA in approximately half the samples was not amplified (Fig. 3). Conventionally, a linker adaptor-mediated PCR (LA-PCR) method [15, 21–23] and a degenerate oligonucleotide-primed PCR (DOP-PCR) method [24, 25] have been used for amplification of DNA segments obtained by chromosome microdissection. In addition, Seifertova et al. [26] reported a different method of chromosome DNA amplification using a different DNA amplification kit. In comparing these methods, the method presented here using the kit has some notable advantages, namely, the ease of sample preparation, shorter amplification time, and lower rate of unexpected amplification of contaminated DNA. On the basis of these features, we believe that the kit is appropriate for amplifying DNA from single microdissected chromosomes of cultivated strawberry. However, in the remaining 144 samples, the amplified DNA segments matched more with the reference genomes of humans, bacteria, nematodes, and other organisms than those of strawberry. The reason for this failure is probably that the microdissected chromosome was not appropriately placed into the 1 µL phosphate-buffered saline (PBS) contained within the PCR tube. In this respect, it is generally very difficult to place a chromosome in the small amount of liquid, and accordingly, there is a necessity to refine this procedure in order to increase the number of samples with amplified DNA segments from microdissected chromosomes. Interestingly, chloroplast and mitochondrial DNAs were rarely amplified using the present method (i.e., organelle DNA sequences constituted approximately 0.21% of the amplified DNA).

### Mapping onto pseudo-molecules of the diploid *Fragaria vesca*, one of the genome donors in cultivated strawberry

**Fig. 4:**
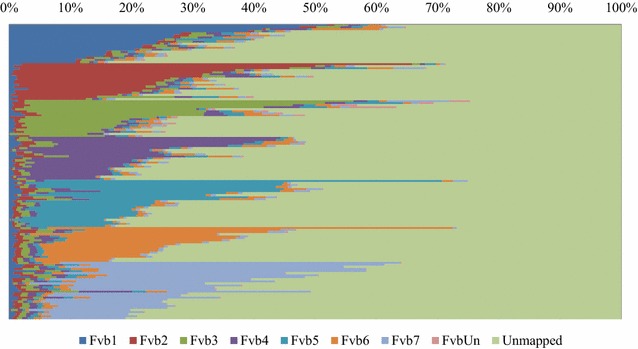
The results of mapping onto pseudo-molecules of *F. vesca* (*2n* *=* *14*), that is one of the genome donors in the cultivated strawberry. The DNA segments that were obtained from a micro-dissected single chromosome, decoded the sequences by the NGS. Every DNA sequence was classified into Fvb1 to Fvb7 of pseudo-molecules in *F. vesca*, FvUn of unassigned sequence in *F*. *vesca* and unmapped one. The percentage of the reads in every pseudo-molecular were calculated, and sorted according to the higher order of the Fvb1 to Fvb7. Then the data were summarized in this figure. Each thin bar exhibited the data of each sample

To examine the similarity of DNA sequences between microdissected single chromosomes and seven different pseudo-molecules (Fvb1 to Fvb7) of *F*. *vesca*, mapping was conducted using the reference genome of *F*. *vesca*, because no pseudo-molecules of the cultivated strawberry have been developed. The 144 sets of sample data were sorted according to the order of the best-matched pseudo-molecule from Fvb1 to Fvb7 (Fig. 4). Among these samples, 19, 18, 18, 21, 23, 17, and 28 samples best matched with Fvb1, Fvb2, Fvb3, Fvb4, Fvb5, Fvb6, and Fvb7, respectively. On the basis of these results, the dissected single chromosomes were identified as homoeologous to the pseudo-molecules in *F*. *vesca*. Furthermore, each sample that was best matched to any pseudo-molecule had 40–80% unmapped reads. However, as shown in Fig. 3, approximately 50–98% of the reads matched with the references genome of strawberry (FAN_r1.1 + *F*. *vesca*). On the basis of these two values, the unmapped reads in Fig. 4 appeared to include some of the reads that originated from the dissected single chromosomes of cultivated strawberry. Thus, it is possible that the assignment of some unmapped reads could be important for clarifying the entire sequence of a single chromosome of cultivated strawberry.

**Fig. 5:**
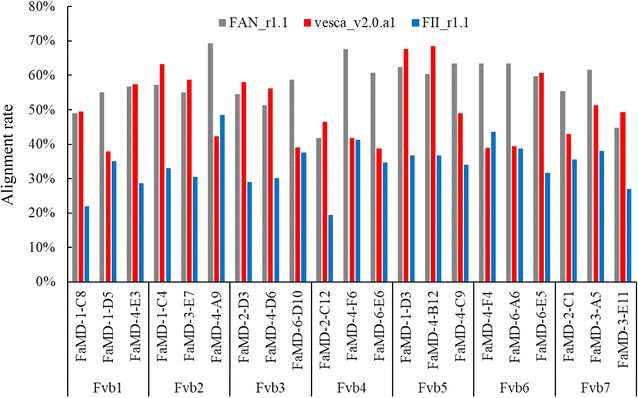
Alignment rates of sequence reads obtained from single-chromosome samples onto the reference sequences of the genomes of *F.* × *ananassa* (FAN_r1.1), *F. vesca* (vasca_v2.0.a1), and *F. iinumae* (FII_r.1.1)

To clarify the source of the unmapped reads, therefore, we investigated the origins of the chromosome samples, since the genome of *F*. × *ananassa* is supposed to contain those of the probable progenitors, e.g., *F. vesca* and *F. iinumae*. A total of 21 samples, three best matched samples from each pseudo-molecule of *F. vesca*, were selected from the 144 samples, and subjected to subsequent sequencing analysis to obtain approximately 4.3 million sequence reads per each sample on average (Additional file 1: Table S1). The reads were mapped on the reference sequences of the genomes of *F.* × *ananassa* (FAN_r1.1), *F. vesca* (vasca_v2.0a1), and *F. iinumae* (FII_r1.1), respectively. Average alignment rates were different among the three references with 58% in *F.* × *ananassa*, 50% in *F. vesca*, and 34% in *F. iinumae* (Additional file 1: Table S1). Expectedly, in accordance with the alignment rate in each sample, it might be possible to distinguish the origins of the chromosome samples into two types (Fig. 5), Fv (alignment rates on *F. vesca* were similar to that on *F.* × *ananassa*) and non-Fv (alignment rates on *F. vesca* were similar to that on *F. iinumae*). The results suggested that homoeologous chromosomes were successfully isolated with our microdissection technique.

**Fig. 6:**
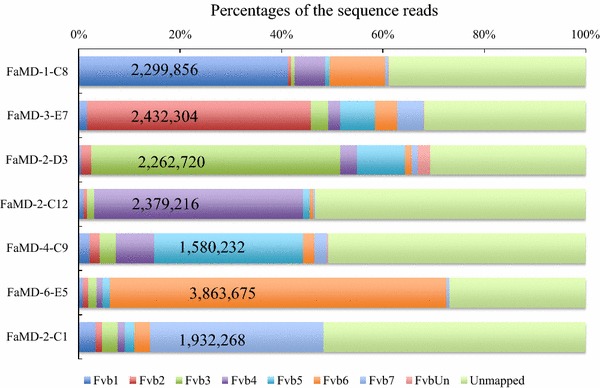
Results of best-matched examples obtained by mapping onto pseudo-molecules of *F. vesca* (*2n* *=* *14*), that is one of the genome donors in the cultivated strawberry. The DNA segments that were obtained from a micro-dissected single chromosome, decoded the sequences by the NGS. Every DNA sequence was classified into Fvb1 to Fvb7 of pseudo-molecules in *F. vesca*, FvUn of unassigned sequence in *F*. *vesca* and unmapped one. In each bar, the number of the reads were exhibited

In order to enable a more complete understanding of the results of the mapping with pseudo-molecules in *F. vesca*, some example results are shown in Fig. 6. The reads of FaMD-1-C8 best matched with approximately 42% of the sequence of Fvb1. Further, FaMD-3-E7, FaMD-2-D3, FaMD-2-C12, FaMD-4-C9, FaMD-6-E5, and FaMD-2-C1 were best matched at approximately 45% for Fvb2, 50% for Fvb3, 40% for Fvb4, 30% for Fvb5, 60% for Fvb6, and 30% for Fvb7, respectively. In each sample, approximately 1.5–4 million reads that matched the specific pseudo-molecule in *F. vesca* were obtained. In contrast, approximately 10–20% of the reads in each sample matched with other pseudo-molecules. Possible explanations for the observed results are as follows. First, the pseudo-molecules might have some incorrectly assigned segments in the sequence because *F. vesca* [27] and *F*. × *ananassa* [9, 14] have 20.74–47.1% repeat sequences in their genomes. Although the assignment of repeat sequences might be problematic, our results indicate that a large percentage of the DNA reads derived from each single chromosome of cultivated strawberry could correspond to a specific pseudo-molecule of *F. vesca*. Second, each chromosome of the cultivated strawberry may contain small segments of the chromosomal DNA derived from each pseudo-molecule of *F. vesca*, based on the evolutionary history of the cultivated strawberry. Third, DNA segments of the other chromosomes might have been contaminated in the PCR tube when the chromosome microdissection was conducted. However, the possibility of such contamination is low, because we precisely microdissected a single chromosome. In addition, the total percentages of the matched reads to the other pseudo-molecules exceeded the level of potential contamination.

### Coverage rate of the amplified DNA from single chromosomes of cultivated strawberry

**Fig. 7:**
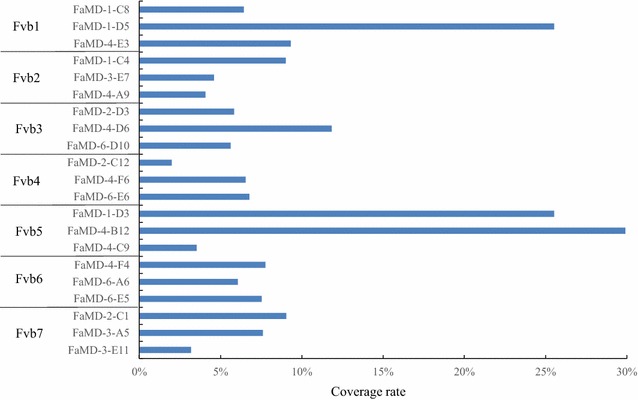
The coverage rate of the reads obtained from a single chromosome DNA in cultivated strawberry. The cover rate was calculated by the following formula. Coverage rate (%) = covered sequence length/reference length of each pseudo-molecular × 100

To determine the percentage of the pseudo-molecules that were covered by the DNA sequence amplified from a single chromosome of cultivated strawberry, the coverage rate was determined for three sample data sets in each pseudo-molecule of *F. vesca* (Fig. 7). The coverage rates varied from 3 to 29.9%. Although the coverage rates of the three samples were greater than 20%, those of the remaining 18 samples were less than 12%. This finding could be explained by the possibility that some chromosomes might have been partially microdissected or that the amplification might have been biased for some reason. In addition, the wide variation may be related to genome composition. As indicted in some papers [7–10], these models specified the presence of just two copies of the *F. vesca*-affiliated A (or Av) subgenome. Thus, if a chromosome in the A (or Av) genome was microdissected and amplified the coverage rates might increase but decrease in the other subgenomes. Currently, it is difficult to clearly determine the cause; however, the coverage rate could be as high as 30% for a single chromosome. On the basis of these results, if the 30% coverage rate were to be applied to each chromosome sample, it would be unnecessary to perform microdissection of many chromosomes to elucidate the entire DNA sequence of each chromosome. For this reason, the experimental method must be improved to increase the coverage rate. However, the entire DNA sequence that corresponds to chromosomal DNA in cultivated strawberry may be revealed through chromosome microdissection using the micromanipulation system.

## Conclusions

The present study was conducted to determine the effectiveness of sequence analysis using single chromosomes for a typical allopolyploid species, cultivated strawberry. Then, the new efficient method to amplify DNA segments from a microdissected single somatic chromosome were exhibited. In addition, it confirmed that the amplified DNA segments were derived from the chromosome of strawberry plants by sequence analysis. The coverage rates of the DNA sequences from the single chromosome onto all pseudo-molecular sequences of the diploid *F. vesca* genome varied from 3 to 29.9%. On the basis of these results, we believe that whole-genome analysis of allopolyploid plants can be enhanced using methodology that employs microdissected single chromosomes.

## Methods

### Chromosome slide sample preparation

Some newly propagated plants of a Japanese octoploid strawberry ‘Reiko’ that was grown in a greenhouse condition were used for the experiment. Pretreatment and fixation of root tips were conducted using a modified version of the method described by Iwatsubo and Naruhashi [28, 29], Nathewet et al. [4, 5], and Yanagi and Noguchi [30]. Root tips were collected at 17:00, pretreated with 2 mM 8-hydroxyquinoline solution for 1 h at approximately 20 °C, and subsequently maintained in the same solution at 4 °C for 15 h until 09:00 the following morning. The root tips were then fixed in a 1:3 (v:v) solution of acetic acid and ethanol for 40 min at room temperature. The fixed roots were trimmed to 2–3 mm from the tip and were softened using an enzyme cocktail, containing 4% cellulase Onozuka RS (Yakult Co. Ltd., Tokyo), 0.3% pectolyase Y-23 (Seishin Pharmaceutical Co. Ltd., Tokyo), 2.1% macerozyme R10 (Yakult Co. Ltd., Tokyo), and 1 mM EDTA pH 4.2 at 37 °C for 25 min. Subsequently, the roots were rinsed twice in distilled water. Then one root tip that was selected using a Pasteur pipette with distilled water and placed in the center of a glass slide. After eliminating the water, 10 µL 45% acetic acid was placed on the root tip, followed by incubation for 2 min and then maceration using forceps. A cover slip was placed on the preparation, tapped gently with a chopstick, heated using an alcohol lamp for a few seconds, and then pressed with a thumb. The glass slide was exposed to −80 °C for at least 5 min in an ultra-low temperature freezer, and then the cover slip was removed using a razor blade at room temperature. The slide samples were dipped in a 70% alcohol solution at −20 °C prior to microdissection.

### Chromosome microdissection

Chromosome microdissection was conducted under a light microscope (BX51; Olympus Co.), which was equipped with a micromanipulation system (MN-4 and MMO-203; Narishige Co.) and a long focus objective lens (× 50 SLMPLN; Olympus Co.). A glass needle for picking up a single chromosome of the cultivated strawberry was fabricated using a glass puller device (PC-10; Narishige Co.). Chromosome microdissection was conducted in a clean room to avoid DNA contamination by atmospheric microorganisms. Single chromosomes on the sample slide were selected individually. After confirming the presence of a single chromosome at the tip of the glass needle under the light microscope, it was placed in a PCR tube containing 1 µL 1 × PBS buffer. The tip of the glass needle to which the microdissected single chromosome was adhered was then pressed against the bottom of the PCR tube and folded, and both were placed in the PCR tube. In total, 310 sample PCR tubes with single chromosomes were prepared from 10 somatic cells of cultivated strawberry.

### DNA amplification and analysis by NGS

DNA amplification was conducted using an Illustra Single Cell GenomiPhi DNA Amplification kit (GE Healthcare Co.), according to the manufacturer’s protocol. After adding 1 µL lysis buffer, the PCR tube was heated at 65 °C for 10 min. To the PCR tube, 11 µL reaction buffer, 1 µL enzyme mix, 1 µL amplification mix, and 4 µL sterile water were then added. The PCR tube was incubated at 30 °C for 180 min, and subsequently heated at 65 °C for 10 min to inactivate the enzymes. Following amplification, the concentration of the DNA was measured using a fluorometer (Qubit^®^ 3.0; Thermo Fisher Co.). The DNA was then fragmented using a DNA Shearing Tube g-TUBE (Covaris, Woburn, MA, USA) or NEBNext dsDNA Fragmentase (New England Biolabs, Hitchin, UK) into lengths of approximately 600 bp for sequencing library preparation using a TruSeq Nano DNA Sample Prep Kit (Illumina, San Diego, CA, USA). The nucleotide sequences of the libraries were determined using a MiSeq system (Illumina) in paired-end mode (301-base) or a NextSeq 500 system (Illumina) in paired-end mode (151-base). The sequence reads were submitted in the DDBJ Sequence Read Archive under the accession number DRA005991.

### Data processing and mapping

Low-quality sequences were removed and adapters were trimmed using PRINSEQ [31] and fastx_clipper in the FASTX-Toolkit (http://hannonlab.cshl.edu/fastx_toolkit). Sequence similarity searches of 1000 randomly selected reads from each library were performed against the NCBI nt (non-redundant nucleotide sequences) database (http://www.ncbi.nlm.nih.gov), the *F. vesca* genome, v2.0.a1 [9], the cultivated strawberry genome, FAN_r1.1 [14], the *F. vesca* chloroplast genome (Accession number NC_015206), and the *A. thaliana* mitochondrion genome (Accession number NC_001284) using the BLASTN program with an *E* value cutoff of ≤ 1*e* ^−10^ [32]. Furthermore, all of the filtered reads were mapped onto the *F. vesca* genome (version v2.0.a1) and the cultivated strawberry genome (FAN_r1.1) as reference sequences using Bowtie 2 [33]. The resulting sequence alignment/map format files were converted to binary sequence alignment/map format (BAM) files. Genome coverage was calculated from the BAM files using the BEDtools script genomeCoverageBed [34].

## Additional file

**Additional file 1: Table S1.** Numbers and alignment rates of sequence reads obtained from single-chromosome samples.
