# Supplementary material for: Sequence analysis of cultivated strawberry (Fragaria × ananassa Duch.) using microdissected single somatic chromosomes
Source: Plant Methods. 2017 Oct 30;13:91. doi: 10.1186/s13007-017-0237-8 (PMC5663147; doi:10.1186/s13007-017-0237-8)
Supplement: Supplementary file 1 [file 13007_2017_237_MOESM1_ESM.docx]

Supplementary Table S1 Numbers and alignment rates of seqeunce reads obtained from single-chromosome samples

Sample name	#Sequence reads	#Reads after quality filtering	FAN_r1.1		vesca_v2.0.a1		FII_r1.1	
			#Reads mapped on the reference	Alignment rate	#Reads mapped on the reference	Alignment rate	#Reads mapped on the reference	Alignment rate
FvMD-1-C8	5,869,990	5,805,983	2,840,726	49%	2,493,100	50%	1,038,230	22%
FvMD-1-D5	4,133,704	4,113,872	2,264,618	55%	1,151,712	38%	1,054,606	35%
FvMD-4-E3	3,322,738	3,271,447	1,854,941	57%	1,407,796	57%	613,798	29%
FvMD-1-C4	2,443,130	2,416,948	1,383,451	57%	1,149,738	63%	491,390	33%
FvMD-3-E7	6,318,824	6,278,066	3,450,428	55%	2,982,040	59%	1,472,316	31%
FvMD-4-A9	3,351,514	3,322,150	2,303,653	69%	990,200	42%	1,160,170	48%
FvMD-2-D3	5,150,314	5,110,541	2,790,585	55%	2,480,524	58%	1,138,062	29%
FvMD-4-D6	2,914,496	2,896,231	1,486,145	51%	1,184,598	56%	559,224	30%
FvMD-6-D10	4,125,542	4,111,342	2,416,332	59%	1,133,764	39%	1,099,254	38%
FvMD-2-C12	5,786,676	5,749,905	2,399,330	42%	2,286,678	46%	895,562	19%
FvMD-4-F6	3,671,968	3,660,571	2,476,513	68%	1,110,298	42%	1,085,146	41%
FvMD-6-E6	2,833,084	2,808,931	1,705,392	61%	734,912	39%	631,972	35%
FvMD-1-D3	3,179,018	3,158,561	1,969,718	62%	1,686,518	68%	759,752	37%
FvMD-4-B12	3,590,178	3,577,955	2,159,076	60%	1,961,066	68%	900,910	37%
FvMD-4-C9	5,434,626	5,380,215	3,409,613	63%	2,177,514	49%	1,436,664	34%
FvMD-4-F4	3,652,708	3,632,243	2,305,886	63%	961,744	39%	1,105,862	44%
FvMD-6-A6	4,674,012	4,643,269	2,946,228	63%	1,337,558	39%	1,295,564	39%
FvMD-6-E5	6,878,880	6,845,941	4,088,564	60%	3,383,322	61%	1,632,704	32%
FvMD-2-C1	6,199,212	6,167,448	3,419,875	55%	2,122,020	43%	1,701,866	36%
FvMD-3-A5	2,373,560	2,348,854	1,445,662	62%	835,964	51%	559,446	38%
FvMD-3-E11	4,486,358	4,468,810	1,998,307	45%	1,782,942	49%	937,610	27%
